# Deep Reinforcement Learning for End-to-End Local Motion Planning of Autonomous Aerial Robots in Unknown Outdoor Environments: Real-Time Flight Experiments

**DOI:** 10.3390/s21072534

**Published:** 2021-04-04

**Authors:** Oualid Doukhi, Deok-Jin Lee

**Affiliations:** 1 Center for Artificial Intelligence & Autonomous Systems, Kunsan National University, 558 Daehak-ro, Naun 2(i)-dong, Gunsan 54150, Jeollabuk-do, Korea; doukhioualid@kunsan.ac.kr; 2 School of Mechanical Design Engineering, Smart e-Mobilty Lab, Center for Artificial Intelligence & Autonomous Systems, Jeonbuk National University, 567, Baekje-daero, Deokjin-gu, Jeonju-si 54896, Jeollabuk-do, Korea

**Keywords:** autonomous navigation, collision-free, deep reinforcement learning, unmanned aerial vehicle

## Abstract

Autonomous navigation and collision avoidance missions represent a significant challenge for robotics systems as they generally operate in dynamic environments that require a high level of autonomy and flexible decision-making capabilities. This challenge becomes more applicable in micro aerial vehicles (MAVs) due to their limited size and computational power. This paper presents a novel approach for enabling a micro aerial vehicle system equipped with a laser range finder to autonomously navigate among obstacles and achieve a user-specified goal location in a GPS-denied environment, without the need for mapping or path planning. The proposed system uses an actor–critic-based reinforcement learning technique to train the aerial robot in a Gazebo simulator to perform a point-goal navigation task by directly mapping the noisy MAV’s state and laser scan measurements to continuous motion control. The obtained policy can perform collision-free flight in the real world while being trained entirely on a 3D simulator. Intensive simulations and real-time experiments were conducted and compared with a nonlinear model predictive control technique to show the generalization capabilities to new unseen environments, and robustness against localization noise. The obtained results demonstrate our system’s effectiveness in flying safely and reaching the desired points by planning smooth forward linear velocity and heading rates.

## 1. Introduction

Research and development in respect of unmanned aerial vehicles (UAVs) have increased dramatically in recent years, particularly in respect of multirotor aerial vehicles, due to their agility, maneuverability, and ability to be deployed in many complex missions. Based on this, the research community has mainly focused on autonomous navigation applications where the environment is static and mapped, and obstacle locations are assumed to be known in advance. Moreover, the accessibility of Global Navigation Satellite System (GNSS) information helps a UAV’s autonomous navigation. However, nowadays, there is an expanding demand for complex outdoor applications, such as rescue, search, and surveillance. In this type of task, the absence of GNSS and environment knowledge due to their dynamics makes it mandatory to use the aerial robot’s exteroceptive sensors for navigation and collision avoidance.

The constraints mentioned above are present in most point-goal autonomous navigation and collision avoidance (PANCA) scenarios. Earlier methods have tackled this issue by dividing it into two modules: a global planning module, which creates trajectories from the robot’s current position to a given target-goal [1]; and path following module, which keeps the robot close to the planned path [2]. However, both modules depend on environment characteristics and robot dynamics, making them sensitive and needing them to be re-tuned for each scenario. Nevertheless, these methods suffer from the stochastic dynamic behavior of the obstacles and high computational cost when applied to highly unstructured environments.

More recently, the success of deep learning (DL) in solving artificial intelligence problems [3] has motivated researchers in the field of autonomous systems to apply recent algorithms to common robotic issues like navigation, decision making, and control [4]. However, DL algorithms work in a supervised fashion, and use structured datasets to train models that require a lot of data and manual labeling, which is a time-consuming process. Reinforcement learning frameworks have been merged with DL to address these limitations, which has led to a new research area called deep reinforcement learning (DRL) [5]. DRL automates the process by mapping high-dimensional sensory information to robot motion commands without referencing the ground-truth (unsupervised manner). It requires only a scalar reward function to motivate the learning agent through trial-and-error experiences of interacting with the environment by seeking to find the best action for each given state. Even though significant progress has recently been made in the DRL area and its application to autonomous navigation and collision avoidance problems [6], existing approaches are still limited mainly to two aspects: (i) some of the algorithms require billions of interactions with the environment, which can be costly. They need very sophisticated computing resources, and the obtained policies are prone to failure in many PANCA scenarios where a GPS signal is not available, which makes them inapplicable in real robotic systems [7]; (ii) methods suffer from the generalization capability to new unseen environments or target goals. These limitations degrade the performance of the navigation system in complex and unstructured scenes.

This work addresses the above-motioned issues by proposing an onboard actor–critic deep reinforcement learning (DRL) approach that allows safe goal navigation by mapping exteroceptive sensors, robot state, and  goal information to continuous velocity control inputs, which allows better generalization and learning efficiency. The proposed method has been evaluated for different tasks: (1) generalization capabilities to new unseen goals, where the mission objective is to navigate toward a target goal that was not seen during training; (2) robustness against localization noise, where a Gaussian noise was added to the robot localization system during the testing phase before moving to the real-world experiments; (3) optimal motion, where the developed approach was compared with a nonlinear model predictive control (NMPC) technique, in terms of path shortness toward the goal; (4) sim-to-real generalization, where the obtained policies were tested in a real aerial robot to demonstrate the navigation efficiency.

In summary, a novel navigation system for a micro aerial vehicle (MAV) has been proposed. To train and test the developed approach, we created a simulation framework based on the Gazebo open-source 3D robotics simulator. The reality gap was closed by simulating the aerial robot and the sensors using the original specifications. A vast set of simulation experiments shows that the proposed approach can achieve point-goal navigation in optimal paths, outperforming the NMPC method. The developed algorithm runs in real-time onboard the UAV using an NVIDIA Jetson board TX2 GPU. The obstacle detection was performed using Hokuyo 2D lidar measurements. The UAV state estimation was achieved by using Intel’s tracking camera RT265. The remainder of this paper is organized as follows. Section 2 introduces related work. Section 3 describe the aerial robot platform, and the architecture of the developed algorithm. Section 4 presents the simulation results. Section 5 presents the real-time experiments, and we finally conclude in Section 6.

## 2. Related Work

Moving from an initial position to another target location is an ordinary task for humans. For a robot, such a job is a significant challenge due to the environment dynamics, especially in respect of aerial robotics. Several works have been proposed for autonomous navigation, and collision avoidance when the obstacles’ location or an environment map are known in advance based on the simultaneous localization and mapping (SLAM) algorithm [8]. Under this assumption, the collision-free trajectory can be computed offline. In the literature, many different approaches exist that perform the path-planning task, for instance, the road-map approach and its different methods [9], artificial potential field approach [10], and other graph-search-based approaches, such as the $A^{*}$ (AStar) algorithm and breadth-first search (BFS) [11].

Those algorithms are highly dependent on the environment map representation method (metric or feature-based). The map’s accuracy and the number of obstacles have a significant influence on the path-finding algorithm. One of our approach’s primary advantages compared to these methods is that it does not require a prior map of the environment or obstacle location. Other classical approaches rely on predefined landmarks that are used on the run time for navigation [12]. Our method does not make any assumption on the landmarks of the environment. With the recent development in edge computing systems, alternative approaches have been proposed for reactive planning, where the system relies on the immediate perception of its surrounding environment for decision-making. This increases the autonomy of the robotic systems and makes them avoid dynamic obstacles. For instance, in [13], the authors proposed an approach based on nonlinear model predictive control (NMPC) for dynamic collision avoidance for a multi-rotor unmanned aerial vehicle. The presented technique combines the optimal path planning and optimal control design into a unified optimization problem; it was tested only in simulation, and it does not consider the model uncertainty and external disturbances. Another work applies an adaptive NMPC for quadrotor navigation, while taking into account specific exogenous signals [14]. This technique seems to be computationally heavy due to the combined online parameter estimation and safe trajectory generation. Ref. [15] presents an NMPC-based strategy for a quadrotor UAV in a 3D unknown environment. This approach is still limited because it assumes that the obstacle is static, which is not the case in the real world. These MPC-based approaches are prone to failure and are vulnerable to the local minima problem.

Attractive alternative approaches for robot motion planning are based on machine learning techniques. Typical methods are supervised imitation learning [16] and deep reinforcement learning [17]. Imitation learning uses expert demonstrations that are saved as datasets for training the navigation policies. The work in [18] showed that it is possible to navigate an autonomous UAV in a forest-like environment by imitating human control. The work presented in [19] extends this approach to an indoor environment where a quadrotor learns to cross a corridor. In [20], the authors explored the application of imitation learning for an unmanned ground vehicle (UGV). However, these algorithms suffer from generalization capabilities to scenarios not included in the training data, which are primarily held for flying robots in 3D environments. Moreover, collecting useful, expert demonstrations for aerial robots is a nontrivial task as these robots can be hard to control, need an expert pilot, and cannot operate for a long time due to the power limitations.

More recently, deep reinforcement learning (DRL) has been used in many robotic applications [21]. For instance, in [22], the authors proposed a proximal policy optimization approach for fixed-wing UAV attitude control. Other work has used the same algorithm for quadrotor UAV attitude control [23]. In [24], navigation of an autonomous underwater vehicle (AUV) was addressed using the deep deterministic policy gradients (DDPG) algorithm. Other work has used the same technique (DDPG) for landing a quadrotor in a moving object [25]. Ref. [26] discusses asynchronous off-policy updates for learning robotic manipulation tasks. Regarding the DRL application for navigation and collision avoidance tasks, there are few works that have addressed these issues. In [27], the authors reported that deep RL-based navigation could be useful in crowded spaces by modeling human–robot and human–human interactions as a reinforcement learning framework. The learned socially cooperative policies were tested on a real ground robot. A differential drive mobile robot point stabilization problem was addressed in [28] by adopting the DDPG algorithm for calculating the desired velocities while taking both kinematic and dynamic constraints into account, such as speed and acceleration limits. Ref. [29] presented a map-based DRL for mobile robot navigation by formulating the obstacle avoidance task as a DRL problem based on a generated local probabilistic cost map, which was treated as an input for the dueling double deep Q-network (DQN) algorithm. This technique is prone to failure in crowded environments where the map is unreliable or unavailable.

However, DRL algorithms’ applications for aerial robotic navigation tasks are still in the early stages of development. In [30], the authors proposed a learning method called CAD2RL. It takes RGB images as its input and generates velocity commands. The policy was trained using the Monte Carlo policy evaluation algorithm. Memory-based DRL for obstacle avoidance in an unknown environment was presented in [31], and the work considers a UAV performing a random exploration in an indoor environment, which is relatively easy compared to a point-goal navigation task. In [32], the authors presented a motion planning technique for a quadrotor UAV. The approach uses a depth image as the input for the DQN algorithm and returns a discrete set of actions to guide the UAV. This approach is still limited; it lacks the generalization capabilities toward new target goals, and the discrete action can create undesirable osculation, which makes the aerial robot drift from the desired trajectory. The work in [33] addressed this limitation by adopting the DDPG algorithm with continuous action space for UAV navigation in 3D space, yet still in an unrealistic simulated environment. The work in [34] applies a DQN algorithm for a drone delivery task, where the UAV tries to reach a predefined goal while avoiding obstacles based on depth images. This approach’s main drawback is that it uses a discrete action space for guiding the UAV, and it was tested only in simulation. Other recent works have applied actor–critic architecture to achieve UAV autonomous navigation in large-scale complex environments, and this was tested only in a simulated environment [35].

## 3. Robot Platform and System Description

**Figure 1:**
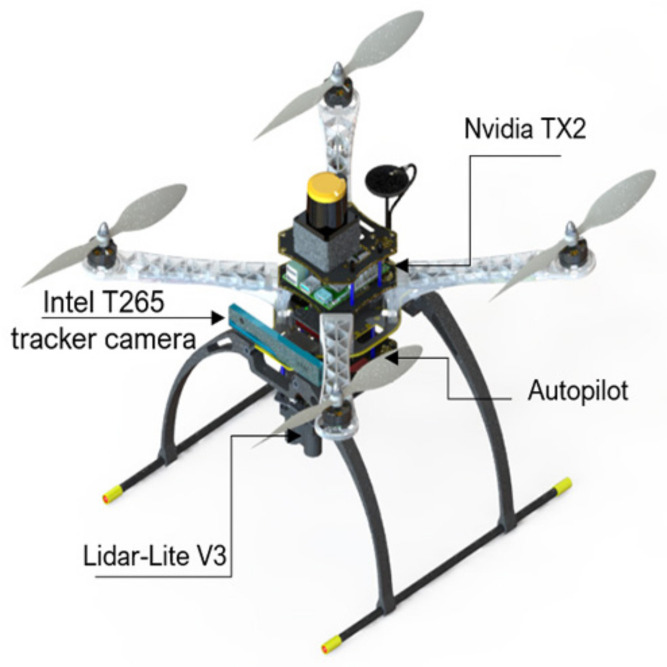
CAD design for the custom-built micro aerial vehicle (MAV).

**Figure 2:**
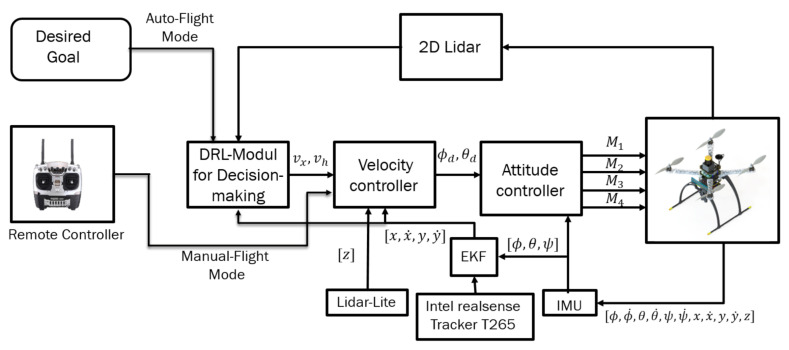
The complete system architecture.

Experiments were performed using a customized quadrotor MAV (Figure 1). The aerial robot includes an autopilot for flight control and an Nvidia Jetson TX2 onboard computer, mounted on top of the Auvidea J120 carrier board. The developed algorithms use a Hokuyo UST-10LX laser scan measurement within the field of view of 90 degrees and the MAV’s position, ground velocity, and orientation, which was estimated using a forward-facing fish-eye Intel tracking camera T265 module fused with other sensors (e.g., IMU data) using an extended Kalman filter (EKF) running on Pixhawk open-source flight control software [36]. For accurate altitude feedback, the system uses a Lidar-Lite V3 laser range finder. A software part relay on the JetPack 3.2 (the Jetson SDK) and a robot operating system (ROS kinetic) were also installed for sensor interfacing. The deep reinforcement learning (DRL) module plays an essential role by adjusting the MAV’s linear velocity $v_{x}$ and heading rate $v_{\psi }$ towards the goal point while avoiding possible obstacles in the path. The detailed configuration of the aerial robot is shown in Table 1. The commanded velocities serve as a set-point for the high-level flight velocity controller. The complete architecture of the proposed system is presented in Figure 2. The human pilot can select between two flight modes, manual or auto, using a radio transmitter. If the auto mode is selected, the DRL module will guide the drone towards the predefined goal-point while avoiding obstacles, and this makes the drone fully autonomous and able to navigate in an unknown outdoor environment. In case of an emergency, the pilot can intervene at any time by switching to manual flight mode.

### Problem Formulation

**Figure 3:**
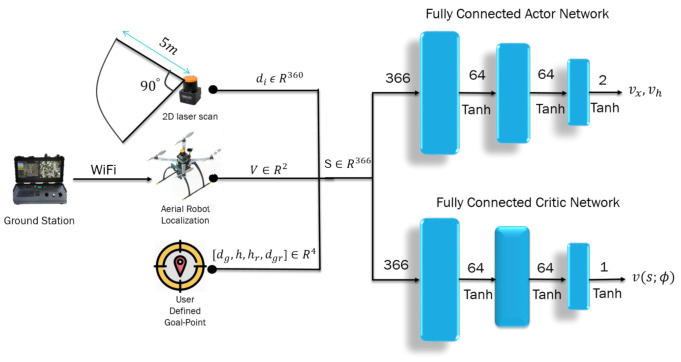
Deep-reinforcement-learning-based goal-driven navigation: the selected state is laser scan measurements $d_{i}$, robot velocities *V*, distance, and heading from the goal point, and their rate of change $\left[d_{g},h,d_{gr},h_{r}\right]$. The outputs are the commanded velocities scaled to $0\leq v_{x}\leq 1$ and $-0.8\leq v_{h}\leq 0.8$, and also to the value function v(s;ϕ).

Conventional autonomous navigation and collision avoidance methods require prior knowledge of the environment for decision making (e.g., obstacle location assumed to be known, availability of the environment map). In an outdoor scenario, the environment is continuously changing, and building an accurate map representation in such cases becomes difficult and unfeasible. The point-goal autonomous navigation and collision avoidance (PANCA) have been formulated as a Markov decision process (MDP) to overcome these limitations. The MAV seeks to find the goal in a forest-like scenario by interacting with the environment using a lidar range finder. The MAV interacts with the environment by performing a continuous action $a_{t}\in A^{2}$, which includes two moving commands (forward velocity and heading rate $v_{x},v_{h}$), and the environment provides a reward scalar r∈R at time t=0,1,2,…,T. to show how good or bad the taken action was in a particular state $s_{t}\in S^{366}$. These interactions can be represented by a tuple $\tau =(s_{0},a_{0},r_{1},s_{1},a_{1},\;\ldots \;,s_{T})$, where $s_{T}$ is the terminal state. In the PANCA task, the MAV reaches the terminal state when it finds the goal-point within 2 m of accuracy, when it crashes into an obstacle, or when the maximum number of steps is reached. To solve this MDP problem, we proposed a learning-based actor–critic architecture that learns the optimal navigation policy $\pi_{\theta }^{*}$, which is parameterized by the weights of the actor neural network θ. The actor-network is designed to map the input states represented by the MAV’s current linear velocities $v_{x},v_{y}$, current distance, and heading from the goal-point; and their rate of change and the laser scan data to a probability distribution over all possible actions. The critic-network approximates the action-value function, as shown in Figure 3.

Both neural networks are the same, containing an input layer with a size of 366, followed by a hidden layer with 64 neurons. Finally, the output is forwarded to the last layer to generate the velocity commands $v_{x},v_{h}$ in the body frame of the MAV. The Tanh activation function follows each layer. The proposed algorithm is a model-free, on-policy, actor–critic, policy-gradient method, in which we try to learn a policy π(θ) by maximizing the true value-function $v_{\pi_{\theta }}$ directly by calculating the gradient of the objective function J(θ) with respect to the neural network’s weights θ.

(1) $$\begin{array}{c} J\left(\theta \right)=E_{s_{0}∼p_{0}}\left[v_{\pi_{\theta }}\left(s_{0}\right)\right] \end{array}$$

(2) $$\begin{array}{c} \nabla_{\theta }J\left(\theta \right)=\nabla_{\theta }E_{s_{0}∼p_{0}}\left[v_{\pi_{\theta }}\left(s_{0}\right)\right] \end{array}$$

To calculate the gradient, we take a full interaction trajectory τ, which can be represented as

(3) $$\tau =s_{0},a_{0},r_{1},s_{1}\ldots s_{T-1},a_{T-1},s_{T},a_{T}$$

The G(τ) function below represents the sum of all rewards obtained during the course of a trajectory τ within *T* step

(4) $$\begin{array}{c} G\left(\tau \right)=r_{1}+\gamma r_{2}+,\ldots ,+\gamma^{T-1}r_{T} \end{array}$$

(5) $$\begin{array}{c} G\left(\tau \right)=\sum_{t=\tau }^{T}\gamma^{\tau -t}r_{\tau } \end{array}$$

where γ is the discount factor. Then we can calculate the probability $P(\tau |\pi_{\theta })$ of the trajectory τ given policy $\pi_{\theta }$ as follows

(6) $$\begin{array}{c} \begin{array}{c} P\left(\tau |\pi_{\theta }\right)=P_{0}\left(s_{0}\right)\pi \left(a_{0}|s_{0};\theta \right)P\left(s_{1},r_{1}|s_{0},a_{0}\right)\\ \ldots P(s_{T},r_{T}|s_{T-1},a_{T-1}) \end{array} \end{array}$$

The gradient of the objective function J(θ) is

(7) $$\begin{array}{c} \nabla_{\theta }E_{\tau ∼\pi_{\theta }}\left[G\left(\tau \right)\right]=\nabla_{\theta }E_{s_{0}∼p_{0}}\left[v_{\pi_{\theta }}\left(s_{0}\right)\right] \end{array}$$

Using the score function gradient estimator, we can estimate the gradients of the expectation $\nabla_{\theta }E_{\tau ∼\pi_{\theta }}\left[G\left(\tau \right)\right]$ as follows:

(8) $$\begin{array}{c} \nabla_{\theta }E_{\tau ∼\pi_{\theta }}\left[G\left(\tau \right)\right]=E_{\tau ∼\pi_{0}}\left[\nabla_{\theta }logP\left(\tau |\pi_{\theta }\right)G\left(\tau \right)\right] \end{array}$$

(9) $$\begin{array}{c} \nabla_{\theta }E_{\tau ∼\pi_{\theta }}\left[G\left(\tau \right)\right]=E_{\tau ∼\pi_{\theta }}\left[\sum_{t=0}^{T}\nabla_{\theta }log\pi \left(A_{t}|s_{t};\pi_{\theta }\right)G\left(\tau \right)\right] \end{array}$$

where $A_{t}$ is the advantage that can be estimated using the n-step λ-target generalized advantage estimation (GAE) as follows:

(10) $$\begin{array}{cc} A_{t}\left(s_{t},a_{t};\phi \right) & =G_{t}-v\left(s_{t};\phi \right)\\ A_{t}^{1}\left(s_{t},a_{t};\phi \right) & =r_{t}+\gamma v\left(s_{t+1};\phi \right)-v\left(s_{t};\phi \right)\\ A_{t}^{2}\left(s_{t},a_{t};\phi \right) & =r_{t}+\gamma r_{t+1}\gamma^{2}v\left(s_{t+2};\phi \right)-v\left(s_{t};\phi \right)\\ A_{t}^{n}\left(s_{t},a_{t};\phi \right) & =r_{t}+\gamma r_{t+1}+\ldots \gamma^{n}v\left(s_{t+n};\phi \right)-v\left(s_{t};\phi \right) \end{array}$$

As a result, the estimated advantage $A_{t}$ can be expressed with TD-λ as

(11) $$\begin{array}{c} A_{t}^{GAE(\gamma ,0)}\left(s_{t},a_{t};\phi \right)=\sum_{l=0}^{\infty }{\left(\gamma \lambda \right)}^{l}A_{t}^{l+1}\left(s_{t},a_{t};\phi \right) \end{array}$$

in which if λ=0 we can get the one-step advantage estimate

(12) $$\begin{array}{c} A_{t}^{GAE(\gamma ,1)}\left(s_{t},a_{t};\phi \right)=A_{t}^{1}\left(s_{t},a_{t};\phi \right) \end{array}$$

Similarly, if λ=1, the infinite-step advantage estimate will be obtained.

(13) $$\begin{array}{c} A_{t}^{GAE(\gamma ,1)}\left(s_{t},a_{t};\phi \right)=\sum_{l=0}^{\infty }{\left(\gamma \right)}^{l}A_{t}^{l+1}\left(s_{t},a_{t};\phi \right) \end{array}$$

where ϕ is the critic network weight, and γ is the discount factor. To reduce the gradient estimate’s high-variance after an optimization step, we clip the gradient update to be in a specific interval [−ϵ,ϵ] with 0.1≤ϵ≤0.3, which leads to minimal variance.

To successfully perform the desired PANCA task, a hybrid reward function r(t) (shaped: with respect to flight time; sparse: with respect to laser scan data and the current distance from the goal) was designed for training the learning agent. The shaped function motivates the flying robot to reach the goal-point in minimal time, while the sparse function lets it avoid colliding with obstacles, it provides a negative reward $r_{obs}$ if the minimum distance from any obstacle is lower than 1.0m. If the distance from any obstacle is greater than 1.0m and the MAV reaches the goal-point within an accuracy of 2m, a significant positive reward is assigned $r_{goal}$, the training episode finishes, and the MAV is randomly reinitialized to a new position. Hence, if the MAV is still far from the goal, a shaped reward will be given $r_{ft}$. By summing the three rewards, we obtain the final reward function r(t). Algorithm 1 presents the designed reward function in detail. The full workflow for the presented approach is shown in Algorithm 2.
**Algorithm 1** Reward Function Definition r(t)
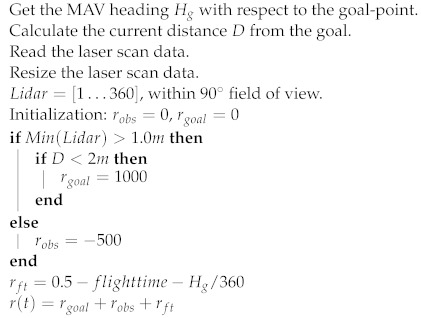
**Algorithm 2** Learning-based control policy for the point-goal autonomous navigation and collision avoidance (PANCA) tasks
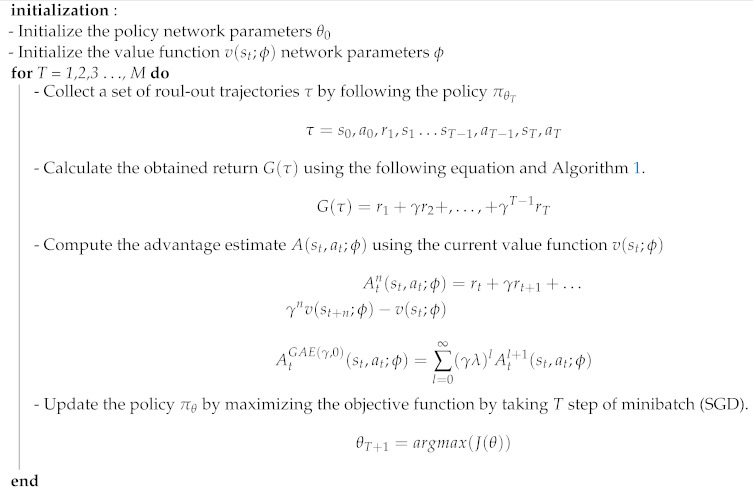


Moreover, to verify the learning-based algorithm’s effectiveness, a comparison with a monlinear model predictive (NMPC) technique has been proposed in simulation experiments. To reduce the computational cost of the NMPC, 10 steps ahead was used for prediction, and the direct multiple shooting method was used to convert the optimal control problem (OCP) into a nonlinear programming problem (NLP). The simplified discrete differentially flat prediction plant model that was used for the MAV is presented below.

(14) $$\begin{array}{cc} x(k+1) & =x(k)+v(k)cos(\psi (k))\\ y(k+1) & =y(k)+v(k)sin(\psi (k))\\ \psi (k+1) & =\psi \left(k\right)+v_{\psi }\left(k\right) \end{array}$$

where $v,v_{\psi }$ are the commanded linear forward velocity and the heading rate, respectively. The NMPC task formulates an optimal control problem (OCP) with constraints on states, manipulated variables, and obstacles that were detected using 2D laser scan measurements, which were considered as a state inequality constraint of a collision-free area. The complete OCP can be formulated as follows:

(15) $$\begin{array}{cc} \min_{X}\; & J=\sum_{h=0}^{T}{\left(X-X_{g}\right)}^{2}P+U_{h}^{2}Q\\ \mathrm{with}\; & U^{T}=\left[U_{1},U_{2}\right]\\ s.t.\; & x(k+1)=x(k)+v(k)cos(\psi (k))\\ \; & y(k+1)=y(k)+v(k)sin(\psi (k))\\ \; & \psi \left(k+1\right)=\psi \left(k\right)+v_{\psi }\left(k\right)\\ \; & X=X_{0}=\left[x_{0},y_{0},\psi_{0}\right]\\ \; & -p_{x}-p_{y}+s_{d}\leq 0\\ \; & v_{min}\leq U_{1}\leq v_{max}\\ \; & v_{\psi_{min}}\leq U_{2}\leq v_{\psi_{max}} \end{array}$$

where $X=\left[x,y,\psi \right],X_{g}=\left[x_{g},y_{g},\psi_{g}\right]$ are the MAV’s current state and the desired goal, respectively; U represents the lumped control inputs $U_{1},U_{2}$; and P,Q are the diagonal weighting matrices as shown in Equation (14).

(16) $$P=\left[\begin{array}{ccc} 1 & 0 & 0\\ 0 & 1 & 0\\ 0 & 0 & 0.1 \end{array}\right],Q=\left[\begin{array}{cc} 0.5 & 0\\ 0 & 8 \end{array}\right]$$

$X_{0}$ is the MAV’s initial state and $p_{x},p_{y}$ is the 2D point cloud reflected from the nearest obstacle from the MAV. $s_{d}$ is the desired safety distance given by the user.

## 4. Training and Testing the Navigation Policy in Simulation

**Figure 4:**
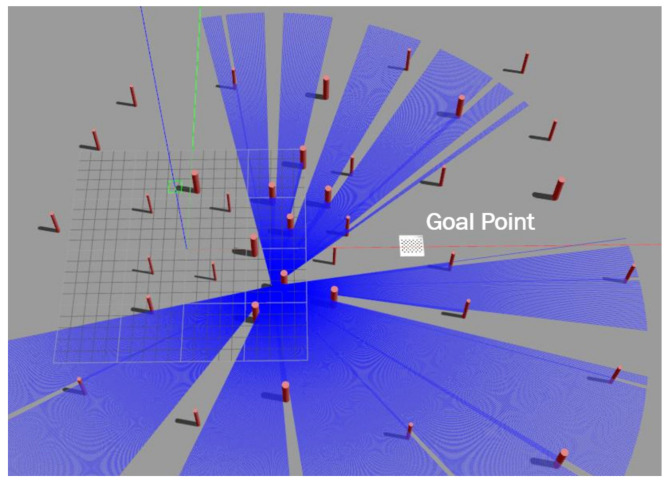
The simulated environment and MAV: The policy was trained in this simulated environment before transferring it to the real world.

The main objective of point-goal navigation is to find the shortest path from a random initial position to the target goal location. To train and test the navigation policy, a simulated outdoor environment that contains multiple obstacles placed randomly was built on top of the Gazebo simulator (see Figure 4). Firstly, training was performed in the environment by generating samples from a single simulated MAV equipped with a localization system and a 2D lidar. The MAV starts from a random initial position and tries to reach the goal. If the MAV arrives at the goal point, it gets a positive reward, both the MAV’s position and the goal position change, and a new episode begins. By doing this, the sample’s correlation will be reduced effectively. The training was performed using an Nvidia GTX 1080Ti GPU, Intel Xeon(R) CPU E5-2650v4 2.20 GHz × 24, and 125 GB of RAM. The MAV’s altitude was 1.2 m and it had a maximum forward speed of 1 m/s and maximum heading rate of 0.8 rad/s. The policy was trained using the Pytorch framework, CUDA 10.0, and CuDNN 7.5. It took approximately 4 days to reach the 8 millionth training step. Table 2 shows the learning parameters in detail.

**Figure 5:**
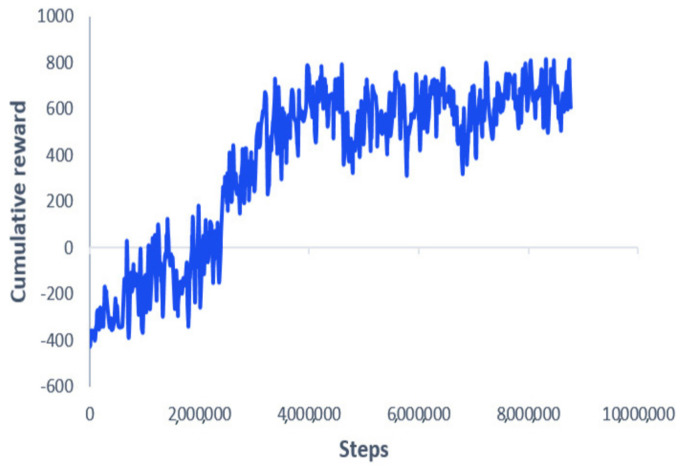
The training curve.

**Figure 6:**
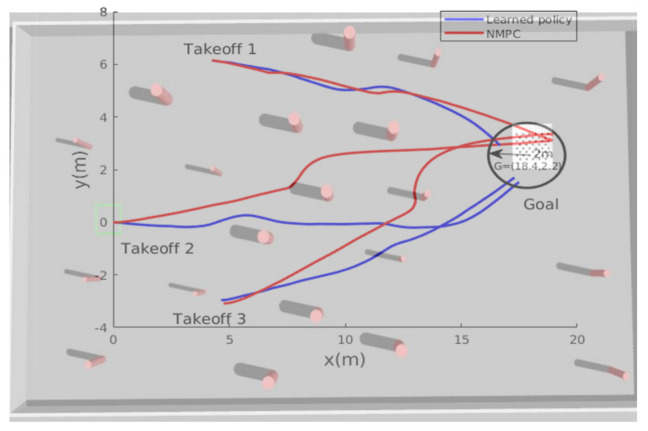
The learned policy vs. nonlinear model predictive control (NMPC): the path taken by the MAV while trying to reach the new goal position starting from 3 different locations.

Figure 5 shows the learning curve. Starting from a negative value of −400, the curve increases gradually until it converges to a particular positive value, around 500. This indicates that the MAV is learning gradually to avoid obstacles and reach the desired goal, leading to positive cumulative rewards. After the training finished, the obtained models were saved for testing purposes. Several performance metrics have been imposed to verify our algorithm’s effectiveness, such as generalization capabilities to new unseen goals, different initial takeoff positions, robustness against localization noise, and motion optimality. In the first simulation tests, the goal and takeoff positions were changed to new places not seen during the training phase. The developed algorithm was benchmarked with the NMPC technique presented earlier. Figure 6 shows the paths taken by the MAV while trying to reach the desired goal location, starting from three different initial positions; both algorithms successfully guided the MAV towards the goal while avoiding the obstacles.

**Figure 7:**
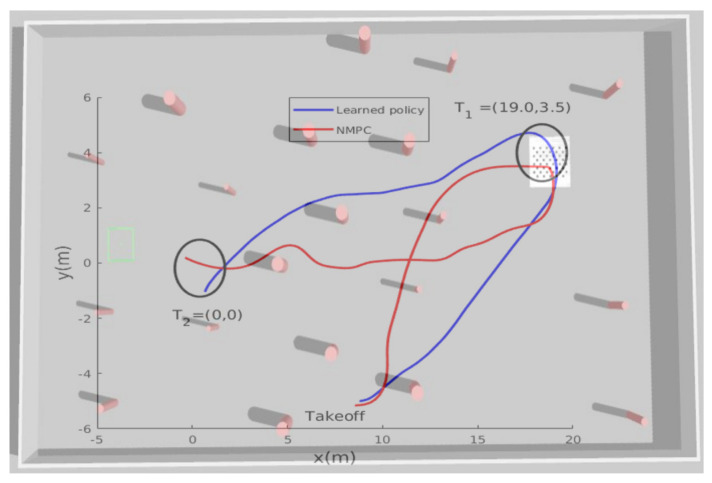
The learned policy vs. NMPC: the path taken by the MAV while trying to reach the new unseen goal positions $T_{1},T_{2}$.

Figure 7 presents the obtained results from the second simulation tests. After takeoff, the MAV was ordered to reach two way-points $T_{1},T_{2}$ within an accuracy of 2 m. In this simulated scenario, the learning-based algorithm showed better performance in terms of trajectory smoothness and shortness compared with the NMPC technique.

**Figure 8:**
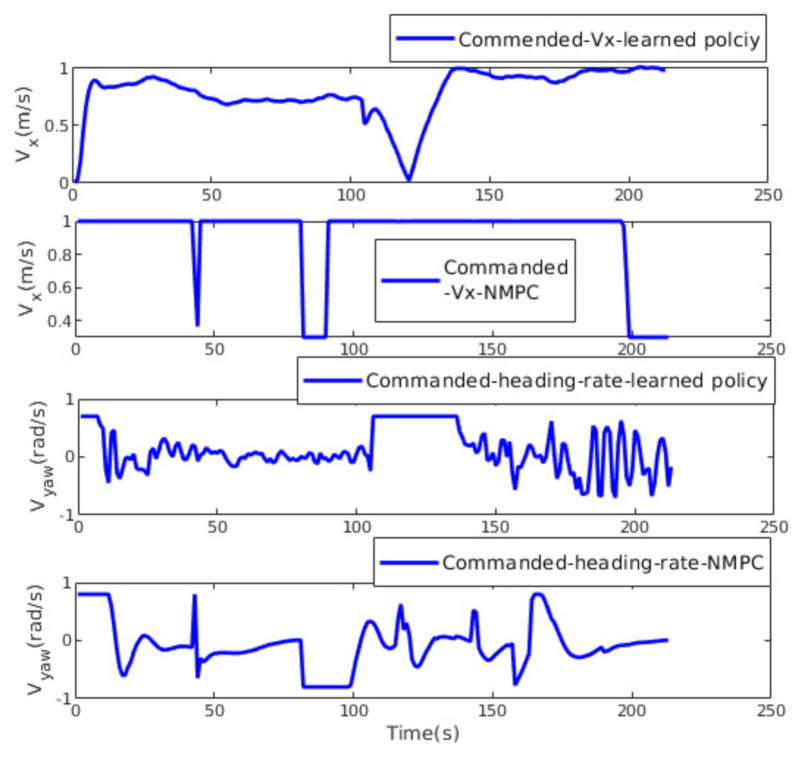
The learned policy vs. NMPC: the commanded heading rate and forward linear velocity.

Samples from the commanded linear and angular velocities are shown in Figure 8. The NMPC control inputs are more oscillating and jerky, which leads to undesirable motion. In contrast, the learned policy shows smooth velocity commands, making it more suitable for the real MAV.

**Figure 9:**
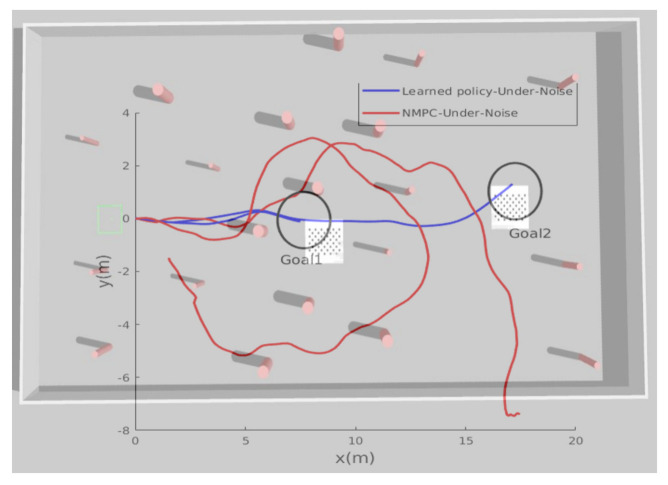
The learned policy vs. NMPC: the path taken by the MAV while trying to reach the new unseen goal position.

In addition to the generalization capability requirements, robustness against noise also was verified by adding Gaussian noise to the localization system (position and velocity) to mimic the real sensor measurements. In this third test, the MAV took off from an initial position of (0,0) under noisy localization input, and was ordered to reach the desired goal (19,20,1.2). Figure 9 shows the current path taken by the aerial robot. The obtained results show that the NMPC could handle the localization noise at all; even with a small variance of σ=0.2, the MAV failed to find the goal and at the end it crashed. However, the new proposed learning-based algorithm shows better results. Under the same simulated noise, the MAV avoided the obstacles and achieved the goal with an accuracy of 2 m. Another test was carried out in the same environment where the desired goal was closer (9,−1,1.2) and relatively easy to find; the NMPC was still not able to guide the MAV towards it, while the learned policy was able to guide the MAV to reach the goal smoothly and robustly. To understand the simulated scenarios, the reader is encouraged to watch the videos included in the Data Availability Statement.

## 5. Real-Time Flight Experiments

**Figure 10:**
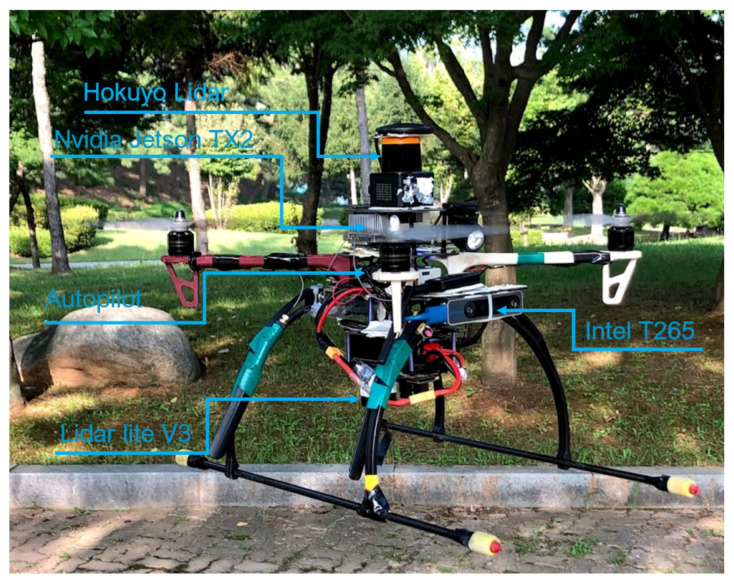
Real hardware platform used for the flight tests.

After validating our simulated scenarios, the obtained trained models were deployed in a real MAV without tuning. Three real-time flight test scenarios were conducted in an outdoor dense forest environment on a sunny day with a wind speed up to 1 m/s. The aerial platform we used is shown in Figure 10. The drone’s onboard flight controller allows control of the MAV through high-level desired velocity commands generated from the learning-based guidance system. Due to the computational limitation, the maximum forward velocity was limited to 1 m/s, and the yaw angle rate of change to 0.8 rad/s, as in the simulation.

### 5.1. Scenario 1: Sim-to-Real Generalization

**Figure 11:**
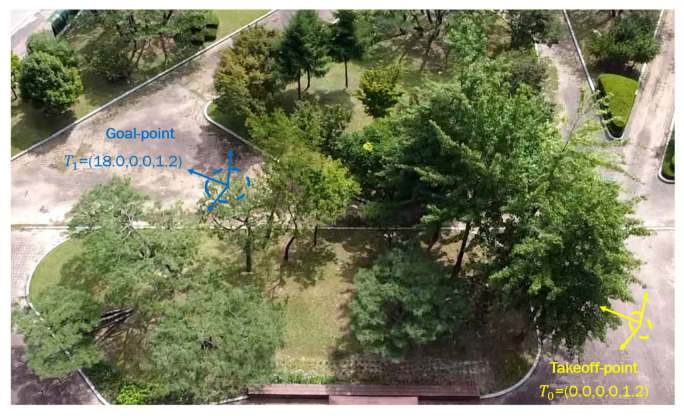
The test environment for scenario 1: after takeoff from $T_{0}$ try to reach $T_{1}$ within 2 m accuracy then go back to the initial position.

**Figure 12:**
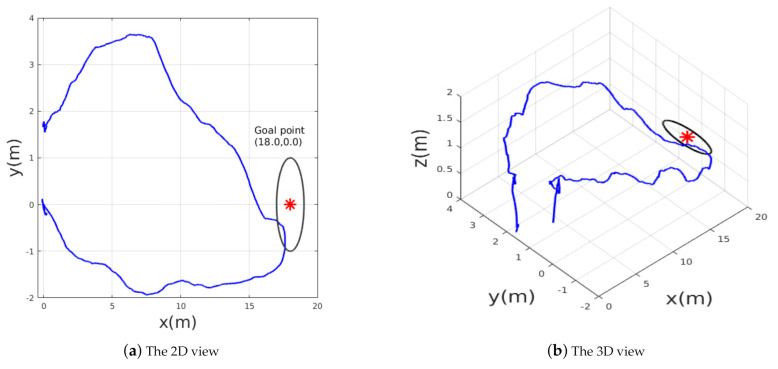
The path followed by the MAV while flying towards the goal and going back to the home point.

The mean objective of this flight test was to validate the trained policy on a real hardware system; Figure 11 shows the test environment where the MAV was commanded to reach a goal point $T_{1}=\left(18.0,0.0,1.2\right)$, which was seen already in simulation; this means that the policy was trained to reach the same goal point in a simulated environment. After reaching the desired goal, the MAV was ordered to go back to the initial takeoff home point $T_{0}=\left(0.0,0.0,1.2\right)$. The MAV’s path while trying to reach the goals $T_{1},T_{0}$ is shown in Figure 12. The obtained results show that the trained policy was able to guide the MAV towards the goal $T_{1}$ within an accuracy of 2m while avoiding colliding with obstacles; once the MAV reaches $T_{1}$, the goal point changes to the home point $T_{0}$, which makes the policy adjust the commanded velocities and head back towards the initial position.

### 5.2. Scenario 2: Motion Optimality

**Figure 13:**
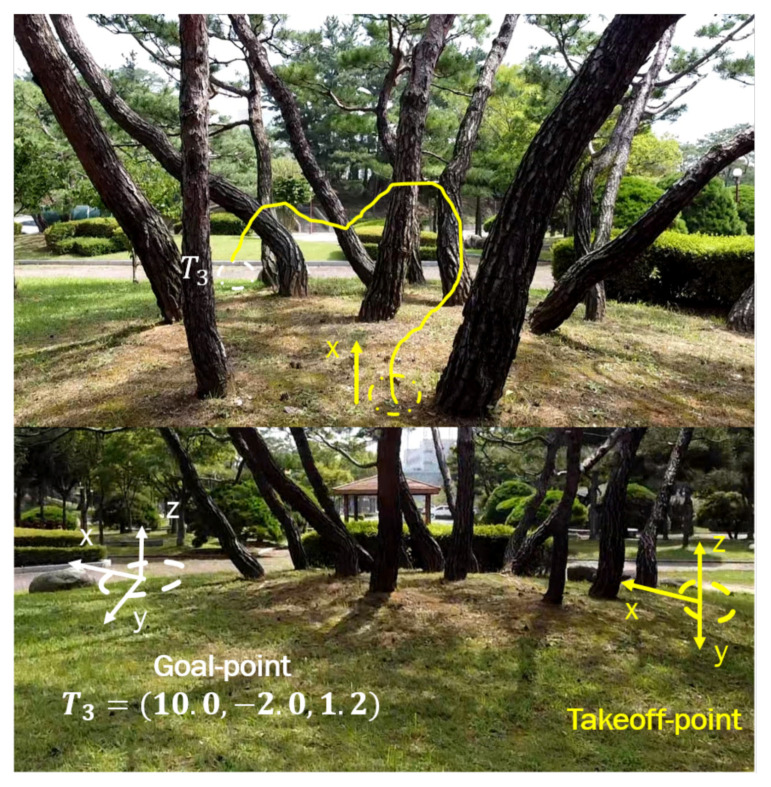
Test environment for the second scenario.

**Figure 14:**
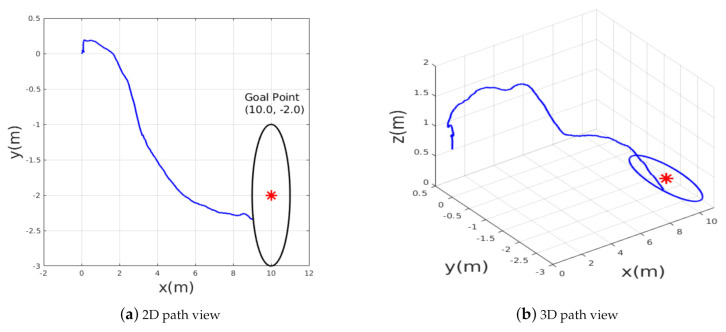
The path taken by MAV in example scenario-2.

To verify the motion optimality, the second test scenario2 has been performed, in which the desired goal point was changed to new location $T_{3}=\left(10.0,-2.0,1.2\right)$ in the presence of more obstacles as shown in Figure 13. The MAV should adjust its heading and forward velocity such that it follows the shortest path towards the goal position while avoiding obstacles. Figure 14 shows the path followed while heading towards the goal point $T_{3}$; the obtained results show that the planned path was near-optimal in terms of shortening the path toward the goal point and the MAV was able to avoid all obstacles and reach the goal within an accuracy of 2 m. The accuracy of reaching the desired goal can be tuned accordingly by setting a certain threshold. If the desired threshold is lower than 2 m, the MAV takes longer to learn the navigation policy. We found that 2 m is the most suitable threshold we can use for this application.

### 5.3. Scenario 3: The Generalization to New Goal Points

**Figure 15:**
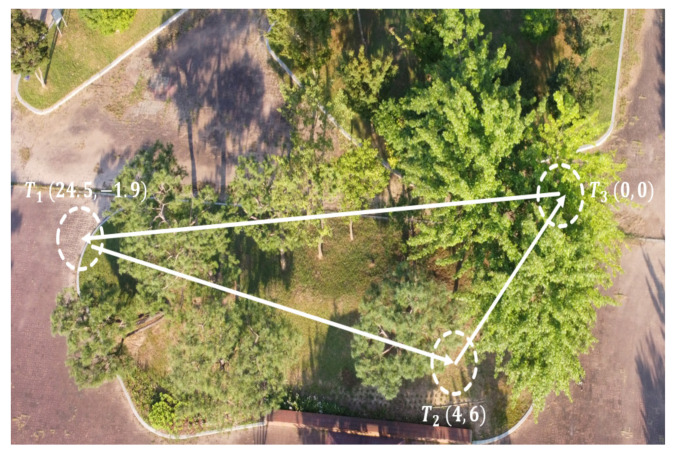
Test environment for scenario 3: multi-waypoint navigation.

**Figure 16:**
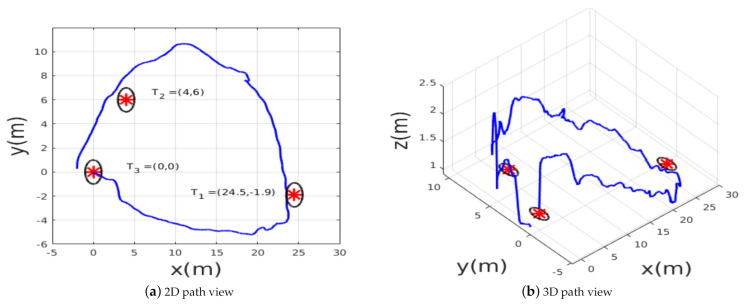
The path taken by the MAV in example scenario 3.

Finally, the last experimental scenario 3 was represented by a medium-range flight, in which the MAV was ordered to reach three waypoints $\left(T_{1},T_{2},T_{3}\right)$ while avoiding obstacles. The desired waypoints are shown in Figure 15. The environment is unstructured and unknown; the trained policy should generate safe velocity commands to reach the points, knowing that these points were not seen during training. Throughout this experiment, the generalization capabilities of the policy can be verified. The path followed by the MAV is shown in the Figure 16, the MAV was able to reach the target points with an accuracy of 2 m while avoiding colliding with the obstacles represented by trees, during this experiment we noticed that the policy always guided the MAV towards the empty space where there were no obstacles then headed towards the goal.

## 6. Conclusions

In this paper we have presented a novel approach for point-goal autonomous navigation and collision avoidance missions for a MAV quadrotor using an actor–critic-based deep reinforcement learning algorithm. The proposed algorithm’s inputs are lidar range finder distance measurements, the MAV’s position, velocity, distance from the target, current heading from the target point, and their rate of change. The navigation policy was entirely trained in a 3D Gazebo-based simulation environment. A comparative study was carried out in simulation with a nonlinear model predictive technique; afterwards, the obtained trained models were deployed on a real MAV platform without any tuning. The simulation and real-world experiments show that the proposed technique was able to guide the MAV towards the desired goal with an accuracy of 2 m in an unknown environment in the presence of localization noise. Future work will address the problem of dynamic obstacle avoidance in 3D space using more sophisticated sensor inputs.

## Figures and Tables

**Table 1:** Aerial robot configuration.

Component	Specs
Hokuyo lidar	270 degrees, UST-10 LX laser scan
Localization camera	Fish-eye camera, Intel T265
Autopilot	Pixhawk V2
Embedded GPU	Nvidia Jetson TX2
Altimeter	Lidar lite V3
Motors	LDPower MT2213-920kv
Propellers	1045MRP
Battery	4S 14.8V, 2200 MAH, 65C
Frame	X Configuration 450 mm

**Table 2:** The learning parameters.

Parameter	Value
γ	0.98
GAE λ	0.95
Trajectory size	2048
Learning rate actor	1 × $10^{-6}$
Learning rate critic	1 × $10^{-5}$
Gradiednt clip ϵ	0.2
Epochs	10
Batch size	128
Max Altitude	1.2 m
Max Forward velocity	1.0 m/s
Max Heading rate	0.8 rad/s

## Data Availability

Videos for the real-time flight experiments can be found at the following link https://doi.org/10.5281/zenodo.4480825 (accessed on 30 January 2021).
